# Effects of T208E activating mutation on MARK2 protein structure and dynamics: Modeling and simulation

**Published:** 2014-09

**Authors:** Sajjad Ahrari, Navid Mogharrab

**Affiliations:** 1 Biophysics and Computational Biology Laboratory, Department of Biology, College of Sciences, Shiraz University, Shiraz, Iran; 2 Institute of Biotechnology, Shiraz University, Shiraz, Iran

**Keywords:** MARK2, Kinase protein regulation, UBA domain, Auto-inhibition, Molecular dynamics simulation, Molecular modeling

## Abstract

Microtubule Affinity-Regulating Kinase 2 (MARK2) protein has a substantial role in regulation of vital cellular processes like induction of polarity, regulation of cell junctions, cytoskeleton structure and cell differentiation. The abnormal function of this protein has been associated with a number of pathological conditions like Alzheimer disease, autism, several carcinomas and development of virulent effects of *Helicobacter pylori*ββαβββ

## INTRODUCTION

MARK2/Par1b is a Serine/Threonine protein kinase, structurally related to AMPK/Snf1 subfamily of the CaMK (Ca^2+^-Calmodulin-dependent protein kinases) group of kinases [1, 2]. This protein was originally associated with a class of gene products, regulating the polarity of cells in *C. elegans* [3]. In human, there are four isoforms of MARKs which are best known for their modulatory effect on microtubule associated proteins (MAPs) [2, 4]. They have also been associated with regulation of cell polarity in epithelial and neuronal cells [5-8]. 

 Conserved sequential arrangement of amino-acids in all AMPK subfamily members including MARK2, give rise to N-terminal header (N), catalytic protein kinase domain (CAT), a putative common docking domain (CD), followed by a Ubiquitin-associated domain (UBA), a spacer domain, and a C-terminal tail domain, which includes the kinase associated domain (KA1). In the majority of AMPK subfamily members, kinase core, UBA and KA1 domains are conserved ones [9]. Activation of MARK2 is achieved through phosphorylation of Thr208, by several upstream kinases like LKB1 and MARKK [10, 11].

UBA domain is a globular domain of 40 residues arranged mainly in three α-helices. It is known as the UBA domain due to its sequence homology to the class of ubiquitin-associated proteins [12]. However, in MARK2, the UBA domain has an unusual fold and is attached to the N-lobe of the kinase core. Accordingly and judging by the published structures of mono- or polyubiquitin docked onto UBA domains of other proteins, UBA is not able to interact with ubiquitin [13, 14]. Although there are several functional role associated with other domains of the MARKs [12, 15], the role of UBA domain is not clearly defined yet [16, 17]. 

Here, we modeled the inactive core structures of native and T208E mutated forms of human MARK2 protein, covering residues 49-363. It has been reported that this mutation increases the kinase activity by four fold [11]. Both models were then subjected to 20 ns of molecular dynamics (MD) simulations. Finally, to evaluate the structural and dynamic consequences of this substitution, a comparative study was performed on important parts of the native and mutated structures including N-lobe, C-lobe and UBA domain. The results showed that activating motions chiefly happen in the N-lobe and these motions are highly affected by UBA domain. The results are discussed regarding the alleged mild auto-inhibitory role of the UBA domain. 

## MATERIALS AND METHODS

**Figure 1:**
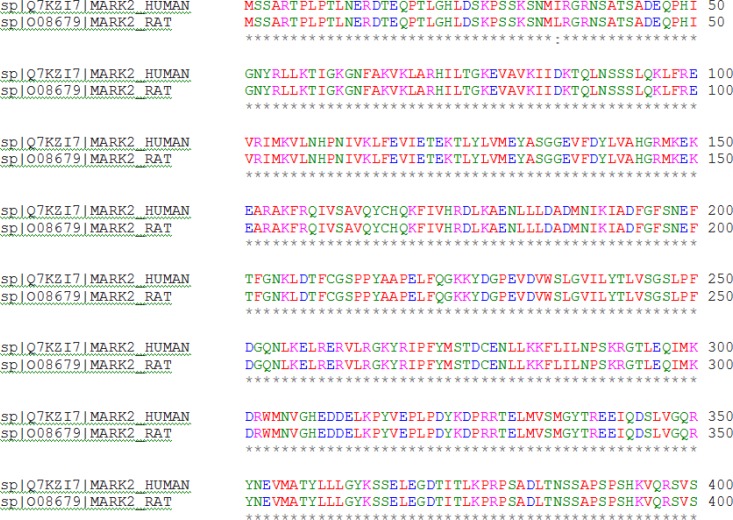
Protein sequence alignment of Human and Rat MARK2 using ClustalX2 version 2.1 [32].

**Modeling**: In order to construct the wild type MARK2 and mutant MARK2_T208E_ structures, MODELLER software version 9.9 [18] was employed using two experimentally-determined structures of inactive MARK2 (PDB: 2WZJ and 1ZMW) as templates. Since most parts of the activation segment are missed in 1ZMW structure, we used the coordinates of 2WZJ structure in order to reconstruct the missing parts. These structures cover residues 49-363 and include kinase core, CD motif and UBA domain. They are all derived from *Rattus* *norvegicus*, but have exactly the same sequence as that of *Homo sapiens* in our target area of study (Fig. 1). Of the 1000 models generated with MODELLER, the one corresponding to the lowest value of the energy and Dope score was selected for further analysis. In order to check for the quality of model, ERRAT [19] and WHATIF [20] software packages were used.

**MD simulation**: All MD simulations were carried out using the GROMACS simulation package version 4.5.5 [21], with Amber force field parameters for energy minimization and MD simulations [22]. The starting atomic coordinates of native and T208E mutated MARK2 was obtained from the modeled structures prepared by MODELLER. Each protein, native or mutated, was centered in a cubic box and immersed in SPC water molecules so that the shortest distance between the protein and the box boundaries was 1.0 nm and periodic boundary conditions were applied. To achieve a neutral simulation box, the net charge of the protein was neutralized by replacing water molecules with Cl^–^ and Na^+^ ions. Each solvated and neutralized system was energy-minimized using the steepest descent algorithm until the maximum force become smaller than 500 kJ/mol.nm. After energy minimization, two separate position-restrained MD simulations were sequentially carried out to equilibrate the solvent and ions around the protein. First, to adjust the system temperature, an NVT MD simulation was performed for 200 ps at 300 K by imposing thermal energy in a constant volume condition using the velocity rescale algorithm (modified Berendsen thermostat) with τ_T_ = 0.1 ps [23]. After arrival at the correct temperature, the resulting atom velocities and coordinates was used to start an NPT MD simulation at 300 K and 1 atm for 200 ps by the Parrinello-Rahman algorithm with τ_P_ = 0.2 ps during which density of the system was stabilized at around 1000 kg/m3 [24]. Finally, the production MD period of 20,000 ps at constant pressure and temperature was performed on native and T208E mutated MARK2, respectively. In all MD simulations the LINCS algorithm was used to constrain all bond-lengths [25]. Lennard-Jones and short-range electrostatic interactions were calculated with 1.0-nm cutoffs, and a particle mesh Ewald algorithm was used for the long range electrostatic interactions [26]. The neighbor list was updated every 10 steps. Each component of the system was coupled separately to a thermal bath, and isotropic pressure coupling was used to keep the pressure at the desired value. A time step of 2 fs was used for the integration of equation of motion.

**Free energy calculations**: Free energy (∆G) of interaction between UBA domain and protein N-lobe was calculated using molecular mechanics Poisson-Boltzmann surface area (MM-PBSA) calculations. 400 frames extracted from the last 8000 ps of each trajectory corresponding to wild and mutant structures were used for analysis. Calculations were performed with the scripts kindly provided by Dimitrios Spiliotopoulos [27].

## RESULTS AND DISCUSSION

**Figure 2:**
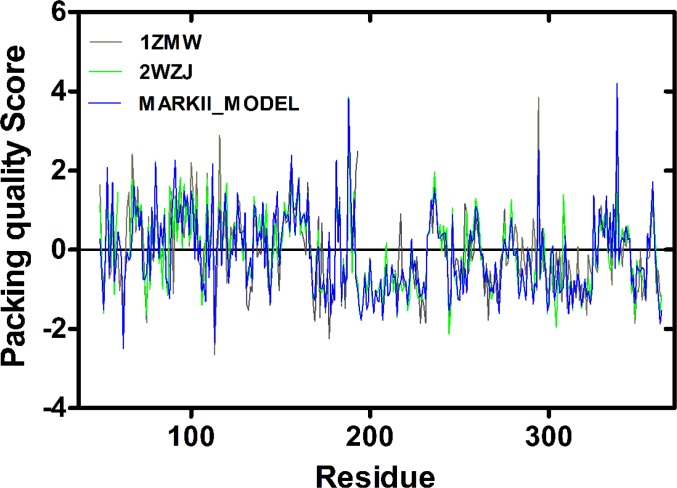
WHAT-IF packing quality score profiles calculated for the templates and modeled structures. The great majority of residues have a score above -2 and none has a score below -4 which is indicative of a high quality model

**Quality of models**: Among the 1000 models generated by MODELLER for MARK2 structure, the one corresponding to the lowest value of the energy and Dope score was selected and quality of the model was evaluated by ERRAT [19] and WHATIF [20] software packages. ERRAT calculates overall quality factor for non-bonded atomic interactions and higher ERRAT score means better quality of the structure. The ERRAT score for templates and the final model were calculated to be 88.3, 95.5 and 86.31, respectively. These values are indicative of high structural quality [19]. We also checked the normality of amino-acid local environment by WHAT-IF program. In order to have a reliable structure, the WHAT-IF packing score should be above -0.5 which is fulfilled in all templates and final modeled structures (Fig. 2).

**Figure 3:**
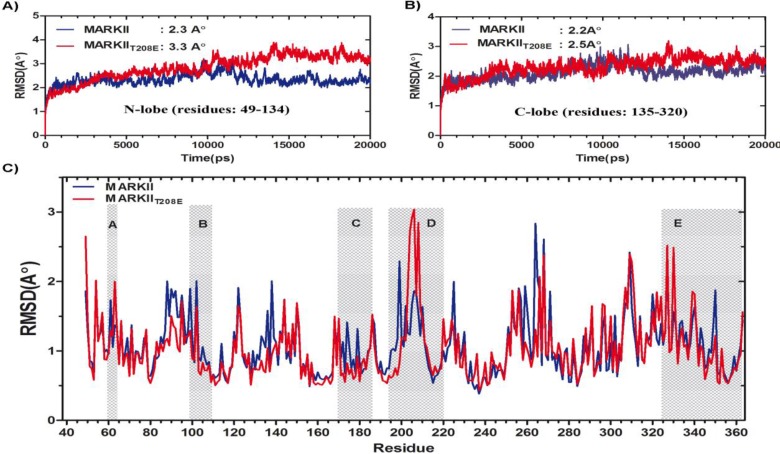
RMSD profiles for the backbone atoms of wild and mutant structures. (A) Time evolution of RMSD for the N-lobe of proteins indicates that the mutant structure is more deviated from the starting structure as compared to the wild type. (B) RMSD measurements for C-lobe residues indicate that structural deviations are similar in the C-lobe of wild and mutant structures (Activation segment was not considered as a part of C-lobe). (C) Backbone RMSD per residue for MARK2 (blue line) and MARK2_T208E_ (red line). The alphabetically highlighted sub-domains are; A: Glycin rich loop, B: αC-helix, C: Catalytic loop, D: activation loop, E: UBA domain

**MD simulation**: Both MARK2 and MARK2_T208E_ models were subjected to 20 ns of MD simulations. The backbone root mean-square deviation (RMSD) of MARK2 and MARK2_T208E_ structures relative to their own starting structures were 1.75 and 2.39 Å, respectively. As seen, average RMSD of MARK2_T208E_ is higher than that of the native one, indicates significant conformational rearrangement in MARK2_T208E_ caused by the activating mutation. For both models, the backbone RMSD as a function of time reaches a relative plateau after about 12 nanoseconds (ns) of simulation (data not shown) and from this time point on, motions have been studied (data not shown). Comparison of RMSD values in the two lobes of the protein implies that gross deviations chiefly happen in the N-lobe of protein and activation segment (Fig. 3).

**Figure 4:**
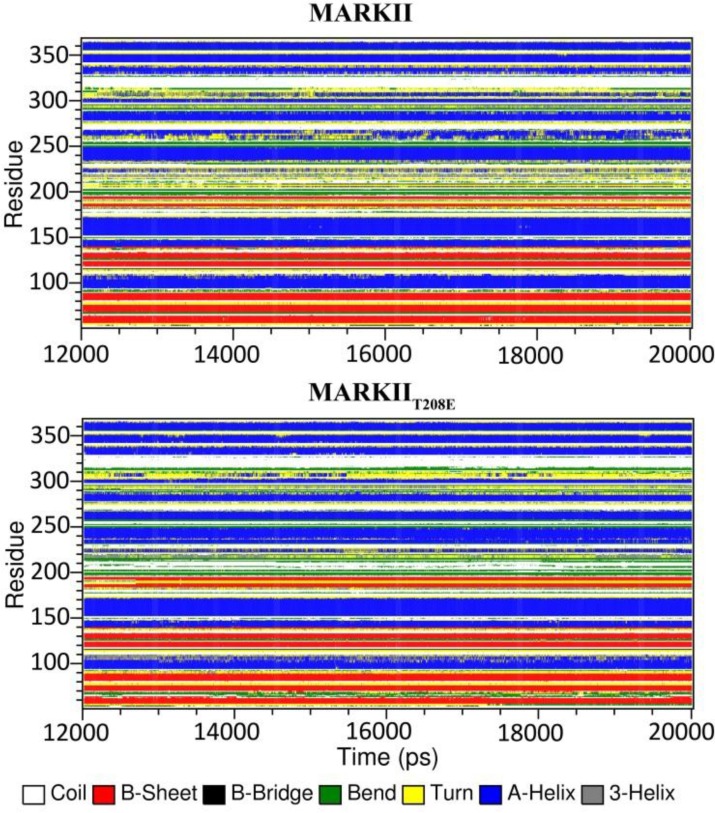
Secondary structure analysis of the native and mutant MARK2 models. Time-dependent secondary structure fluctuations of MARK2 (top panel) and MARK2_T208E_ (lower panel) models calculated for the last 8 ns of simulation using the DSSP program. The occurrence of secondary structure elements is indicated by using a color code. The Stability of β-sheets (residues: 1-80) and αC-helix (residues: 92-106) is reduced within the mutant structure. Activation loop of the mutant model (residues: 193-220) mostly assumes bend and coil structures which is suggestive of a non-stable intermediate structure of this loop. UBA domain secondary structure (residues: 320-363) does not go through any radical changes in both structures

**Figure 5:**
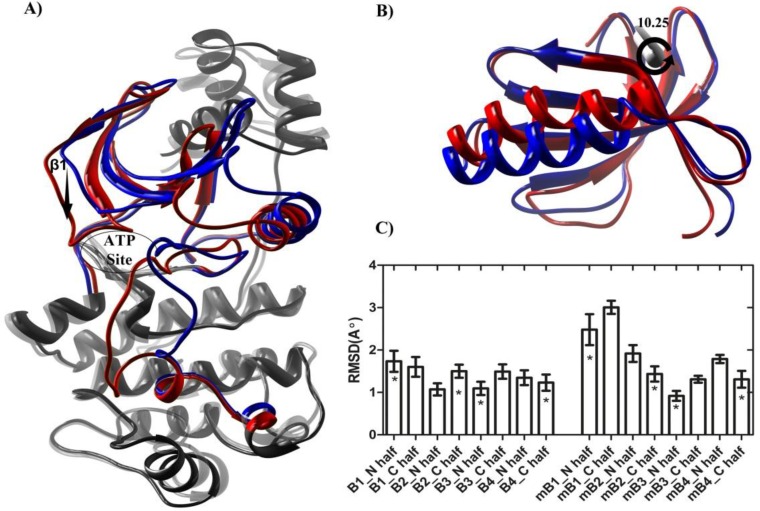
Distinctive structural displacements. (A) Colored zones show the most deviated sub-domains of MARK2 (blue) and MARK2_T208__E_ (red) average structures. Downward movement of β1 in MARK2_T208E_ structure tightens the ATP binding site. (B) Upon mutation, the protein N-lobe rotates by about 10 degrees against the hinge region (rotation angle was measured by UCSF Chimera software version 1.5.3 [33]). (C) RMSD changes for N-half and C-half of N-lobe beta sheets (B1, B2, B3 and B4). UBA neighboring parts (marked by asterisks) are less deviated in mutant structure while in the wild structure, RMSD changes do not follow an ordered pattern (m stands for mutated structure

**Structural changes of N-lobe**: N-lobe of the protein is comprised of five beta sheets giving rise to the so called barrel like structure and a helix (αC-helix) that is proved to have a key role in regulation of numerous kinase proteins activity [28]. Through the activation process, this lobe tilts toward the C-lobe by rotating against hinge region, the zone that connects N-lobe of protein to C-lobe [28]. DSSP analysis shows that through the simulation time, stability of the first three beta sheets residues: 49-88 is reduced within the MARK2_T208E_ structure compared to that of MARK2. This is also the case for αC-helix (residues: 92-105) (Fig. 4). Comparing the average structure of MARK2 and MARK2_T208E_ shows that the N-lobe of the protein has rotated by about 10 degrees toward the C-lobe. This rotation is accompanied by a decrease in the average distance between β1 and β6 sheets (from 17.33Å to 12.51Å) which causes further tightening of the ATP entrance site. As is expected due to T208E mutation, beta sheets of the N-lobe are dragged toward the kinase active site, but nevertheless RMSD measurements indicate that movements of β1-β4 sheets toward the active site are not uniform and UBA-neighboring parts have been less deviated from the starting structure in comparison with those of having no direct interaction with this domain. In the case of wild type (MARK2) structure, RMSD pattern does not show an ordered trend like that of MARK_T208E_ structure (Fig. 5).

**Figure 6:**
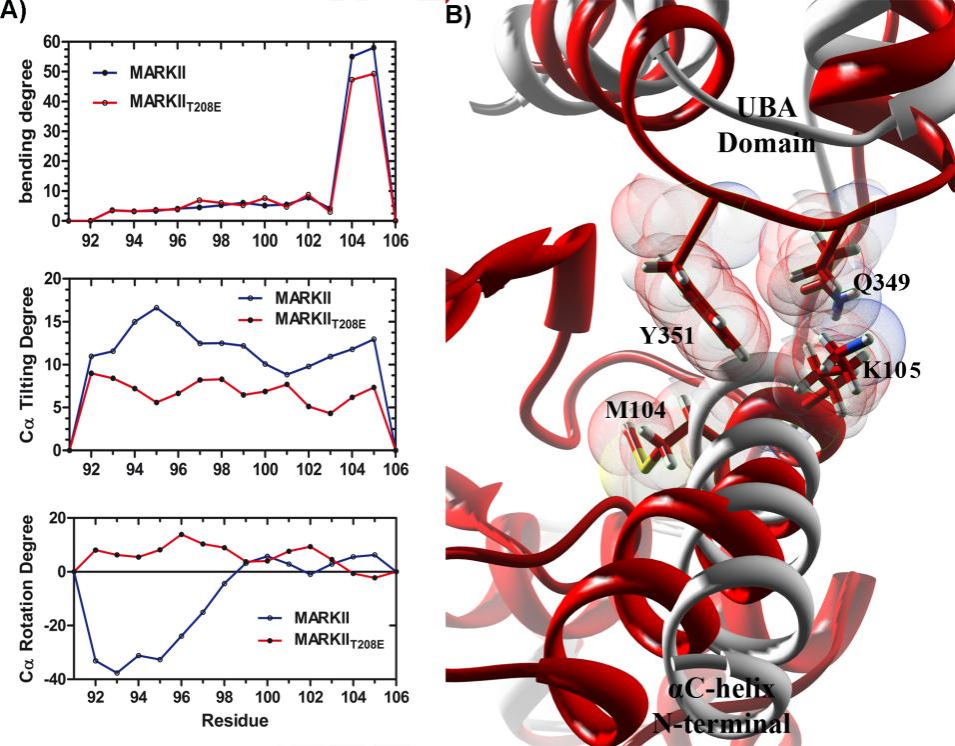
Motions of αC-helix regarding the UBA domain effect. (A) Bending, tilting and rotating motions for αC-helix residues. In mutant structure (red), rotating and tilting motions show a decreasing trend from N-terminal to C-terminal residues. (B) Key Interactions between UBA domain and C-terminal residues of αC-helix in MARK2_T208E_ average structure (red). Superimposing this structure on the starting structure (Light gray) shows that αC-helix is hooked to the UBA domain by C-terminal residues and bends from this point through the simulation time

Activation of protein kinases is associated with the movement αC-helix toward the protein active site, so that several conserved interactions with DFG motif and activation loop can be formed. Restrictions applied on this motion is a common regulatory mechanism among protein kinases [28]. Superimposing the MARK2 and MARK2_T208E_ average structures revealed that although this motion of αC-helix is set out in MARK2T_208E_ structure, but again like that of beta sheets, this expected motion toward the protein active site is not uniform in all through the αC-helix structure (Fig. 6). To analyze αC-helix motions in more detail, we measured the average bending, tilting and rotating motions for C_α_ atoms of this helix, relative to each other. In the case of MARK2_T208E_ structure, analysis of bending motion showed that although Cα atoms from Ser92 to Ile103 follow a similar trend but Met104 and Lys105 have a drastically different bending index. The latter residues give rise to the C-terminus of αC-helix with both having engagement to UBA domain (Fig. 6 and Table 1). It seems that this helix is bent from the point of Met104. For tilting and rotating motions of αC-helix in MARK2_T208E_ structure, the N-half residues show higher tilting and rotating motions. On the other hand , measurement of RMSF for αC-helix shows that the N-half of this helix is comparatively less stable compared to the C-half. It is as if the N-half is pivoting around the C-half (Fig. 3). Considering all these facts, it seems that the residues of N-terminal half have set out toward the active site within the MARK2_T208E_ structure to fulfill their activating motion, but the C-terminal half residues that are close to UBA domain are mostly stuck into this domain, unable to accompany. Although for MARK2 structure a likewise trend can be distinguished, but it is not as ordered as that of MARK2_T208E_ structure (Fig. 3).

**Table 1:** *Existence probability of hydrogen bonds involved *in the interaction of UBA domain with the activation segment residues* during the last 5 ns* of *simulation*

**UBA domain **	**Activation Segment **	***MARK2*** *	***MARK2***_T208E_ *
TYR351-Main chain	VAL118-Main chain	76.51	17.50
TYR351-Side chain	PHE116-Main chain	13.15	-
GLU353-Side chain	ASN52-Side chain	34.57	24.97
GLU353-Main chain	GLU117-Side chain	12.30	11.02
LEU360-Main chain	LYS77-Side chain	25.37	14.40
TYR363-Side chain	GLN130-Side chain	68.32	71.23
GLN349-Side chain	LYS105-Main chain	-	38.87
TYR351-Main chain	VAL118-Main chain	-	67.21
TYR351-Main chain	GLU117-Main chain	-	9.70
**Activation Segment **	**Activation Segment **	***MARK2***	***MARK2***_T208E_
ASP193-Main chain	ASN198-Side chain	67.91	-
ASN198-Main chain	ASP193-Side chain	51.44	60.68
ASN180-Side chain	ASP193-Side chain	47.74	98.73
SER197-Side chain	ASP193-Side chain	22.97	-
GLU199-Main chain	ASP193-Side chain	13.07	99.67
CYS166-Side chain	PHE194-Side chain	33.34	15.25
PHE196-Main chain	PHE194-Main chain	15.17	-
ASP193-Main chain	PHE196-Main chain	-	26.77
PHE200-Main chain	SER197-Side chain	23.72	18.12
LYS177-Side chain	GLU199-Side chain	-	111.95**
LYS205-Side chain	GLU199-Side chain	21.14	-
ASN180-Side chain	GLU199-Side chain	-	100.90
LYS205-Main chain	THR201-Main chain	69.26	-
ASN204-Main chain	THR201-Main chain	29.82	-
LYS205-Side chain	GLU208-Side chain	-	17.55
GLY203-Main chain	THR201-Side chain	-	36.29
**N-lobe **	**Activation Segment **	***MARK2***	***MARK2***_T208E_
ASN63-Main chain	ASN204-Side chain	68.33	-
LYS82-Side chain	SER197-Main chain	44.64	
LYS82-Side chain	ASN198-Main chain	15.95	24.17
LYS82-Side chain	PHE200-Main chain	30.62	77.33
ASN63-Side chain	ASN204-Side chain	70.01	-

* H-bond Occupancy (%) in MARK2 and MARK2_T208E_

** More than one H-bond exists

**Structural changes of C-lobe**: The C-lobe of MARK2 mostly consists of several helices which are stacked on another and a long flexible loop known as activation segment, which has a prominent role in ATP proper positioning and phosphor-transfer reaction [28]. According to the DSSP analysis, the stability of  H-helix (residues: 285-290) and CD-like motif (residues: 305-310) has decreased through the simulation time within MARK2_T208E_ structure. On the other hand, the index of secondary structure for β6, β7, αE-helix (residues: 220-225), αF-helix (residues: 230-240) and α1-helix of UBA domain (residues: 329-335) is more intense within the mutant protein compared to wild structure (Fig 4). Activation loop is another part with noticeable structural deviations as the average structures of wild and mutant forms are compared (Fig. 5). Within all available wild type inactive structures of MARK2, several important residues of this loop are missed from the X-ray structures, probably due to high fluctuation index. Comparative RMSF analysis of the simulated structures is also indicative of relatively high fluctuations of this loop in both wild and mutant structures. Detailed analysis of the MARK2 trajectories shows that along the simulation, this loop folds on itself through a network of hydrogen bonds (Table 1) and mostly takes the structure of turns and bends (Fig. 4). This structure is tethered to the N-lobe of protein and occludes the ATP binding site. Along the simulation of MARK2_T208E_ structure, those interactions related to N-lobe loosen up (Table 1) and this loop moves further away from N-lobe.Actually, the activation loop moves1.5 Å away from the G-loop and αC-helix. This results in more intense fluctuations of this loop compared to the wild structure (Fig. 3). On the other hand, in the N-terminal part of this loop, inter-residue hydrogen bonds weaken, leading to a secondary structure shift from bend and turns to random coils (Fig. 4). Accordingly, conformation of the activation loop becomes more stretched with higher fluctuation index.

**Figure 7:**
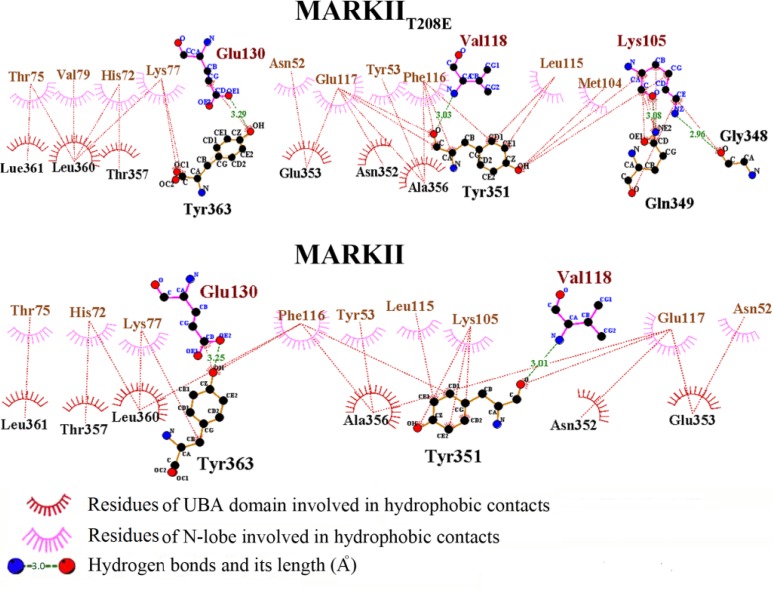
Network of interactions between α3-helix of UBA domain and N-lobe of protein derived from LIGPLOT software analysis [34]. Analysis of the hydrogen bonds and hydrophobic interactions between the UBA domain and protein N-lobe residues implies that this interactive network exists in both wild and mutant structures and is even more prominent within the mutant structure

**Structural changes of UBA domain**: Analysis of the structural deviations for the UBA domain, also suggests a non-uniform trends in localization of three α-helices of this domain. Within the starting structure, the α3-helix is docked against the N-lobe through a network of hydrophobic interactions and hydrogen bonds. Residues like Leu360 and Leu361 from α3-helix interact with Lue73, Val79, Tyr53, Thr75 and Phe116 from N-lobe, while Glu353-Asn52, Thr357-His72, Thr357-Gln117 and Tyr363-Glu130 residue pairs link the two domains by forming a network of hydrogen bonds. Throughout the 20 ns of simulation, a similar story is also repeated within the mutant structure (Fig. 7 and Table 1). This causes the reduction of mass center distance between UBA and N-lobe from 19.65 Å in wild structure to 18.87 Å within the mutated one. In agreement, MM/PBSA calculations indicated that free energy of interaction between the UBA domain and N-lobe of protein reduces from -175 kJ/mol in MARK2 to -184 kJ/mol in MARK2T208E structure, which suggests a more powerful interaction of UBA domain and N-lobe in MARK2T208E structure (Table 2). Further analysis show that while α3-helix of UBA domain keeps resting against N-lobe, the other two helices move away from each other and cause about 160 Å^3^ increase in UBA domain volume and 45 Å^2 ^increase in surface accessibility of MARK2_T208E_ average structure. However, in spite of N-lobe approach toward the protein mass center and decrease of UBA domain distance from the protein N-lobe, these motions are not associated with a significant reduction in the mean distance of atoms from the protein mass center. In agreement, compactness of the protein structure remains almost unchanged in mutant structure (the gyration radius and protein volume were calculated to be 21.27 Å and 58.0 nm^3^ for MARK2 model compared to 21.25 Å and 57.6 nm^3^ for MARK2_T208E_ structure). Detailed analyses imply that dragging movements of UBA and N-lobe domains toward the protein mass center in MARK2_T208E_ structure have been compensated by simultaneous expansion of UBA domain, so that the total protein volume remains almost constant in mutant structure.

Modulation of enzymatic activity through an extension situated to the C-terminal of catalytic core which wraps around the core domain of enzyme is a common regulatory mechanism in many protein kinases [28]. In MARK2, UBA domain is suggested to be of such sort. In the current study, we tried to address this possibility by inducing T208E mutation and analyzing the enzyme behavior through a 20 ns of MD simulation. Our results showed that as is expected for a mutation with a mild activatory function, the protein sets out toward the active structure but the UBA domain neighboring parts fail to set out their motion toward the active state. These results are in agreement with those reports that suggest a mild auto-inhibitory function for this domain. However, reports on the functionality of UBA domain have been contradictory looking and enigmatic although nearly the same approaches have been exploited to study its function [16, 17].

Induction of T208E mutation and studying the protein structure with small-angle X-ray scattering (SAXS) analysis by Marx and colleagues showed that the UBA domain is attached to the N-lobe during the study and no significance difference in protein compactness was observed through the analysis [17]. Regarding the close interaction of UBA domain and N-lobe, they have suggested that this domain could be pulling the N-lobe back and making the catalytic cleft wide open. By removing the UBA domain from the protein, they come to this conclusion that it has a mild auto-inhibitory activity [17]. Our results are relatively in concert with these reports and it looks as if the UBA-neighboring parts of the N-lobe are hooked by the UBA domain and are not easily allowed to accomplish their expected activating motions.

**Table 2:** MM-PBSA binding free energy (kJ/mol) components calculated for the interaction of UBA domain with the protein N-lobe

	**G**_col_	**G**_ps_	**G**_vdw_	**G**_nps_	**G**_binding_
**MARK2**	-135.64 16.3	-3.33 0.3	-51.78 2.1	-5.27 0.1	-175.1 52.6
**MARK2**_T208E_	-126.12 22.7	-1.18 0.2	-54.07 2.3	-5.49 0.1	-184.6 22.8

G_col_: coulombic term,

G_ps_: polar salvation term,

G_vdw_: Van der Waals term,

G_nps_: non-polar salvation term,

G_binding_: computational binding free energy

In a separate study, Jaleel and colleagues came to different results by showing that the enzymatic activity of MARK2 was strikingly reduced after omission of UBA domain [16]. Their results suggest that not only the UBA domain is not having an auto-inhibitory role but its presence is indispensable for the enzymatic activity. They also showed that after the activation, UBA domain leaves the protein N-lobe and rests against C-lobe in a new position. The only difference of their approach seems to be the use LKB1 instead of MARKK as the upstream kinase to phosphorylate and activate MARK2. Their analysis implied that upon activation, the protein radius of gyration was significantly reduced and UBA domain was detached from N-lobe, resting against C-lobe, in a new position [16]. Our findings indicated that although the whole structure shows no compactness upon activation, the UBA domain expands and at the same time the kinase core becomes more compact. Actually it seems that expansion of the UBA domain compensates for compactness of the kinase core, and so the whole protein volume remains relatively unchanged. We also found that the UBA domain steadily remains attached to the N-lobe of protein during the whole time of simulation.

There are also reports indicating that the activity of MARK2 phosphorylated by LKB1 is about 20 times higher than that phosphorylated by MARKK [10, 11]. On the other hand, comparing the enzymatic activity of MARK2 for structures having and lacking the UBA domain and after activation by upstream kinases like MARKK, implies that the kinase activity is slightly higher, when the UBA domain is omitted [17]. Accordingly, it is reasonable to assume that LKB1-MO25-Strad complex neutralizes the UBA domain auto-inhibitory function by detaching it from the protein N-lobe and prone the MARK2 for activity. This supposition is further supported by the fact that the UBA domain is an indispensable part of the interaction between the LKB1-MO25-Strad complex and MARK2. So the observations reported by Jaleel et al. [16] may be justified this way.

There is also another reason to attribute a mild auto-inhibitory function to the UBA domain; Those RD protein kinases that need phosphorylation on activation segment to fulfill their activity, have a basic residue like arginine or lysine on β9 strand which contributes to the formation of RD pocket. Substitution of these residues with neutral or acidic ones, emancipate the kinase from phosphorylation dependency for activation [29, 30]. In MARK2, Asn198 and Glu199 are situated in this position and the location of Asn198 is conserved in all members of the AMPK subfamily of kinases which also hold the UBA or UBA-like domain, except BRSK1 and BRSK2 [10, 16]. It seems that although the ionic interaction of this conserved asparagine with basic residues of αC-helix and HRD motif (RD pocket) can trigger the kinase to achieve its active state (even if it is not phosphorylated), but the UBA domain applies a mild break to this trend and makes the MARK2 activation dependent on phosphorylation of the conserved threonine (Thr208) of the activation loop. The extent of rigidity within the UBA or UBA-like domains may also contribute to the level of auto-inhibition. The sequence of this domain is not highly conserved among all members, but all have a similar 3D structure consisting of three alpha helices (α1, α2 and α3). There is also a conserved glycine residue within the connecting loop of α1 and α2 helices (Gly336 in MARK2) [16]. In MARK2, this glycine seems to contribute to the greater flexibility of the UBA domain and makes the motions of these three helices less dependent on each other. So while the α3-helix is dragged toward the N-lobe through activation, the other two helices can assume new positions. This helps α3-helix motions to be less dependent on the other two helices and weakens the restrictions associated with dynamics and position of α1 and α2 helices. Within the structure of AMPKα1 and AMPKα2, there’s a glutamate in place of this glycine which may results in more rigidity of UBA like structure (AID domain). This may explain the higher kinase activity in AMPKα after the omissions of AID domain compared to kinase activity in MARK2 after omission of UBA domain [31]. Confirmation of these suggestions awaits more biochemical and structural studies.

## References

[1] Manning G, Whyte DB, Martinez R, Hunter T, Sudarsanam S (2002). The protein kinase complement of the human genome. Science.

[2] Drewes G, Ebneth A, Preuss U, Mandelkow EM, Mandelkow E (1997). MARK, a novel family of protein kinases that phosphorylate microtubule-associated proteins and trigger microtubule disruption. Cell.

[3] Guo S, Kemphues KJ (1995). Par-1, a gene required for establishing polarity in C. elegans embryos, encodes a putative Ser/Thr kinase that is asymmetrically distributed. Cell.

[4] Espinosa L, Navarro E (1998). Human serine/threonine protein kinase EMK1: genomic structure and cDNA cloning of isoforms produced by alternative splicing. Cytogenet Genome Res.

[5] Böhm H, Brinkmann V, Drab M, Henske A, Kurzchalia TV (1997). Mammalian homologues of C. elegans PAR-1 are asymmetrically localized in epithelial cells and may influence their polarity. Curr Biol.

[6] Zhang X, Zhu J, Yang GY, Wang QL, Qian L, Chen YM, Chen F, Tao T, Hu HS, Wang T, Luo ZG (2007). Dishevelled promotes axon differentiation by regulating atypical protein kinase C. Nat Cell Biol.

[7] Cohen D, Brennwald PJ, Rodriguez-Boulan E, Müsch A (2004). Mammalian PAR-1 determines epithelial lumen polarity by organizing the microtubule cytoskeleton. J Cell Biol.

[8] Cohen D, Rodriguez-Boulan E, Müsch A (2004). Par-1 promotes a hepatic mode of apical protein trafficking in MDCK cells. Proc Nati Acad Sci USA.

[9] Matenia D, Mandelkow EM (2009). The tau of MARK: a polarized view of the cytoskeleton. Trends Biochem Sci.

[10] Lizcano JM, Göransson O, Toth R, Deak M, Morrice NA, Boudeau J, Hawley SA, Udd L, Mäkelä TP, Hardie DG, Alessi DR (2004). LKB1 is a master kinase that activates 13 kinases of the AMPK subfamily, including MARK/PAR-1. EMBO J.

[11] Timm T, Li XY, Biernat J, Jiao J, Mandelkow E, Vandekerckhove J, Mandelkow EM (2003). MARKK, a Ste20-like kinase, activates the polarity-inducing kinase MARK/PAR-1. EMBO J.

[12] Marx A, Nugoor C, Panneerselvam S, Mandelkow E (2010). Structure and function of polarity-inducing kinase family MARK/Par-1 within the branch of AMPK/Snf1-related kinases. FASEB J.

[13] Ohno A, Jee JG, Fujiwara K, Tenno T, Goda N, Tochio H, Kobayashi H, Hiroaki H, Shirakawa M (2005). Structure of the UBA domain of Dsk2p in complex with ubiquitin: molecular determinants for ubiquitin recognition. Structure.

[14] Varadan R, Assfalg M, Raasi S, Pickart C, Fushman D (2005). Structural determinants for selective recognition of a Lys48-linked polyubiquitin chain by a UBA domain. Mol Cell.

[15] Moravcevic K, Mendrola JM, Schmitz KR, Wang YH, Slochower D, Janmey PA, Lemmon MA (2010). Kinase associated-1 domains drive MARK/PAR1 kinases to membrane targets by binding acidic phospholipids. Cell.

[16] Jaleel M, Villa F, Deak M, Toth R, Prescott AR, Van Aalten DMF, Alessi DR (2006). The ubiquitin-associated domain of AMPK-related kinases regulates conformation and LKB1-mediated phosphorylation and activation. Biochem J.

[17] Marx A, Nugoor C, Müller J, Panneerselvam S, Timm T, Bilang M, Mylonas E, Svergun DI, Mandelkow EM, Mandelkow E (2006). Structural variations in the catalytic and ubiquitin-associated domains of microtubule-associated protein/microtubule affinity regulating kinase (MARK) 1 and MARK2. J Biol Chem.

[18] Sali A, Blundell TL (1993). Comparative protein modelling by satisfaction of spatial restraints. J Mol Biol.

[19] Colovos C, Yeates TO (2008). Verification of protein structures: patterns of nonbonded atomic interactions. Protein Sci.

[20] Vriend G, Sander C (1993). Quality control of protein models: directional atomic contact analysis. J Appl Cryst.

[21] Van Der Spoel D, Lindahl E, Hess B, Groenhof G, Mark AE, Berendsen HJC (2005). GROMACS: fast, flexible, and free. J Comput Chem.

[22] Lindorff‐Larsen K, Piana S, Palmo K, Maragakis P, Klepeis JL, Dror RO, Shaw DE (2010). Improved side‐chain torsion potentials for the Amber ff99SB protein force field. Proteins.

[23] Berendsen HJC, Postma JPM, van Gunsteren WF, DiNola A, Haak J (1984). Molecular dynamics with coupling to an external bath, J Chem Phys.

[24] Parrinello M, Rahman A (1981). Polymorphic transitions in single crystals: A new molecular dynamics method. J Appl Phys.

[25] Hess B, Bekker H, Berendsen HJC, Fraaije JGEM (1997). LINCS: a linear constraint solver for molecular simulations. J Comp Chem.

[26] Darden T, York D, Pedersen L (1993). Particle mesh Ewald: An N⋅ log (N) method for Ewald sums in large systems. J Chem Phys.

[27] Spiliotopoulos D, Spitaleri A, Musco G (2012). Exploring PHD fingers and H3K4me0 interactions with molecular dynamics simulations and binding free energy calculations: AIRE-PHD1, a comparative study. PloS One.

[28] Endicott JA, Noble ME, Johnson LN (2012). The structural basis for control of eukaryotic protein kinases. Ann Rev Biochem.

[29] Johnson LN, Noble M, Owen DJ (1996). Active and inactive protein kinases: structural basis for regulation. Cell.

[30] Nolen B, Taylor S, Ghosh G (2004). Regulation of protein kinases: controlling activity through activation segment conformation. Mol Cell.

[31] Chen L, Jiao ZH, Zheng LS, Zhang YY, Xie ST, Wang ZX, Wu JW (2009). Structural insight into the autoinhibition mechanism of AMP-activated protein kinase. Nature.

[32] Larkin M, Blackshields G, Brown N, Chenna R, McGettigan PA, McWilliam H, Valentin F, Wallace IM, Wilm A, Lopez R (2007). Clustal W and Clustal X version 2.0. Bioinformatics.

[33] Pettersen EF, Goddard TD, Huang CC, Couch GS, Greenblatt DM, Meng EC, Ferrin TE (2004). UCSF Chimera--a visualization system for exploratory research and analysis. J Comput Chem.

[34] Laskowski RA, Swindells MB (2011). LigPlot+: multiple ligand-protein interaction diagrams for drug discovery. J Chem Inf Model.

